# A report from the AAPM Subcommittee on Guidelines for Competency Evaluation for Clinical Medical Physicists in Radiation Oncology

**DOI:** 10.1120/jacmp.v17i4.5804

**Published:** 2016-07-08

**Authors:** Daniel C. Pavord, Steven Birnbaum, Douglas Boccuzzi, Steven deBoer, D. Jay Freedman, Michael Schell, Steven Sutlief

**Affiliations:** ^1^ Radiation Oncology Health Quest Poughkeepsie NY; ^2^ Department of RadiationOncology Swedish Medical Center Englewood CO; ^3^ Radiation Oncology UConn Health Center Farmington CT; ^4^ Department of Radiation Medicine Roswell Park Cancer Institute Buffalo NY; ^5^ Radiation Oncology Riverside Cancer Care Center Newport News VA; ^6^ Radiation Oncology University of Rochester Medical Center Rochester NY; ^7^ Department of Radiation Medicine and Applied Sciences University of California San Diego San Diego CA USA

**Keywords:** competency, credentialing, medical physics, radiation oncology

## Abstract

The goal of this report is to provide a framework from which an institution can develop a competency and credentialing program. It is not intended to be adopted as written, but rather as a list of suggestions from which the institution develops their program. A clear distinction should be made between the initial evaluation of the competency of new staff (credentialing) and the ongoing verification of the competency of existing staff. Furthermore, whenever new technologies are implemented, the entire staff would be subject to the credentialing process. Competencies involve the ongoing verification of the performance of a procedure according to the established policies and procedures at a facility. This can be done by audits of work product, direct observation of performance, self‐evaluation, or testing.

PACS number(s): 87.10.‐e, 87.90.+y

## I. INTRODUCTION AND DEFINITIONS

The evaluation of the initial and ongoing competency of a clinical medical physicist in radiation oncology presents unique challenges whether the physicist is part of a group or practicing as a solo physicist. Though the challenges for each situation are different, the solutions provided below have common processes all with the ultimate goal of ensuring quality performance and that patient safety remains paramount.

The goal of this report is to provide a framework from which an institution can develop a competency and credentialing program. It is not intended to be adopted as written, but rather to serve as a list of suggestions from which the institution develops their program.

A clear understanding of the definition of terms is important in the discussion of this topic. One definition of competence (paraphrased from Wikipedia):^(1)^

Competence is a standardized requirement for an individual to perform properly a specific job. It encompasses a combination of knowledge, skills and behavior utilized to improve performance. More generally, competence is the state or quality of being adequately or well qualified, having the ability to perform a specific role.

The concept is addressed by the AAPM in their Statement on Quality Radiation Therapy:^(2)^

In the United States, medical physicists demonstrate competence in their discipline by obtaining board certification. Certification is a rigorous, multi‐year process that requires considerable clinical experience under supervision and passage of written and oral examinations. Medical physicists follow detailed quality assurance and safety protocols established to insure that cancer treatments with radiation are conducted according to the prescription prepared by the physician for every treatment of every patient. Medical physicists follow guidance from documents developed by the AAPM and in cooperation with other professional societies. The AAPM has numerous committees dedicated to quality assurance and safety in radiation therapy.

While board certification does establish a general level of competence and is a strict requirement for a Qualified Medical Physicist (QMP), board certification alone is not an end point or a certification of competency as defined in this document. In addition, continuing education after receiving certification is an essential component to maintaining certification and demonstrating continued competency.

The AAPM Code of Ethics^(3)^also addresses competency:

“Members must be aware of the limitations of their knowledge, skill, and experience. They shall undertake only work that they are qualified to perform and shall seek additional education and training or consultation when indicated. Members should disclose known limitations in their ability when relevant.“

This important principle should be adhered to in all cases.

For the purposes of this report we consider competency to be defined as having met the local requirements to be able to complete a task independently. Individuals who have the proper credentials (board certification) to perform the task but have not completed the process to be deemed competent by the local institution, are not considered competent. As described below, the process of demonstrating competency could be as simple as providing the proper documentation showing experience with the task and reading the local policies and procedures. This initial determination of competency for a new employee is credentialing. The bottom line is that each institution should establish these processes for each task. Competency is considered a local condition, while qualifications and credentials are a more global concept. Having the requisite knowledge and training is a requirement to be deemed competent, but not an end point. The local competency process must also be completed. Having the proper credentials should not be confused with credentialing, which, again, is a local process where an institution establishes the competency of a new employee. In this report we consider credentials to be the minimum requirements needed to apply for a position and would include items such as degree, completion of a residency, and board certification.

Ultimately the responsibility for evaluating staff for competency and ensuring that new staff is credentialed falls to the chief medical physicist. The question remains, Who is responsible for ensuring the competence of the chief medical physicist or a solo physicist? This could be done in coordination with either the human resources department or the medical staff office depending on the local organizational structure.

## II. SCOPE OF THE REPORT

The goal of this report is twofold:

1. Provide a framework to develop medical physics competencies in a variety of situations, as there are unique features to each and a one‐size‐fits‐all solution would not be practical.
  - Solo physicist
  - Physics group
  - Experienced physicist
  - New graduate
  - Initial competency evaluation (credentialing)
  - Ongoing competency evaluation
  - Assessing competency in a changing or expanding role (e.g., in the implementation of new procedures or technology)
2. To develop a set of criteria for physicians and administrators to evaluate that the necessary components of a medical physics competency program are present and are reviewed annually.

Outside reviews, training, and staffing will be discussed in the context of the aforementioned topics.

### It is not the intent of this report to compile specific descriptions of all needed competencies

A clear distinction should be made between the initial evaluation of the competency of new staff (credentialing) and the ongoing verification of the competency of existing staff. Furthermore, whenever new technologies are implemented, the affected staff would be subject to the credentialing process. Competencies involve the ongoing verification of the performance of a procedure according to the established policies and procedures at a facility. This can be done by audits of work product, direct observation of performance, self‐evaluation, or testing. Having a structured program will benefit the institution and the staff in many ways. First, the documentation can be used to fulfill the requirements of accrediting bodies, such as The Joint Commission.^(4)^ The staff can also use the documentation to establish competency at a new facility should they change positions. The principal benefit, though, comes from ensuring that all staff are competent in the tasks that they are performing. Each step within the radiation therapy process can be a cause of patient harm and, therefore, deserves a rigorous determination of competency which should not be done in an ad hoc manner.

## III. COMPETENCY AND CREDENTIALING FRAMEWORK

### 1. Certification, Maintenance of Certification, and Recertification

All medical physics clinical activities should be performed under the supervision of a QMP.^(5)^ A list of activities is detailed in the AAPM Scope of Practice document.^(6)^ The AAPM regards certification by an appropriate certifying board in the appropriate medical physics subfield as the appropriate qualification for the designation of QMP. Those medical physicists with board certification must follow the maintenance of certification or recertification process of the board under which they are certified in order to remain credentialed. Furthermore, all medical physics staff should document their continuing education and practice quality improvement activities. The American Board of Radiology (ABR) recommends that all diplomates participate in the ABR Maintenance of Certification program and requires it for diplomates with time‐limited certificates.^(7)^ The Canadian College of Physicists in Medicine (CCPM)^(8)^ and the American Board of Medical Physics (ABMP)^(9)^ both issue time‐limited certificates and require ongoing professional practice and continuing education. We support this common principle of the certifying boards, and recommend that all physicists, regardless of certification status, maintain the recommended levels of professional involvement and continuing education.

### 2. Policies and Procedures

In order to establish the proper performance of an activity, there must be a clear understanding of exactly how that activity is expected to be performed. These expectations are contained in detailed policies and procedures at each clinical facility. The policies establish the overall scope of activities, their frequency (if applicable), and tolerances for results (if applicable). The procedures are detailed step‐by‐step descriptions of the activities. Policies and procedures should be developed by the QMP(s) of the department for every physics task.

The policies and procedures should be reviewed at least annually by all involved medical physics staff to ensure familiarity and to update or make improvements to them.

### 3. Credentialing of New Staff

The credentialing of clinical Medical Physics staff should be a three‐step process. The written policies and procedures are reviewed by the new staff with a currently credentialed QMP, the new staff observes the requisite number of procedures, and finally, the new staff member demonstrates competence by performing the activity under the direct supervision of existing staff. Note that additional iterations of this proctoring process may be required for new staff members lacking sufficient prior experience, and a more streamlined process would be appropriate for someone with prior experience with the activity, as described in the Introduction.

The process of credentialing a solo physicist should include a peer review process, as outlined in the AAPM TG‐103 report.^(10)^ In addition, where possible, outside credentialing should be obtained, as there may be no one within the institution with the ability to evaluate competency in certain areas. This may involve, but is not limited to, vendor training for the department's technologies, if available, the use of the Imaging and Radiation Oncology Core (IROC, formerly RPC) phantoms or dose monitoring program, or similar services from IAEA or the University of Wisconsin.^11^, ^12^, ^13^ At a minimum, a dry run of the activity, including appropriate in‐phantom measurements, should be done and all results presented to an outside peer. The medical director should also review the results to ensure that the clinical goals are met and that any limitations are understood. These concepts are recommended for all sites, but are imperative in a solo physicist environment. The practice accreditation programs can be an excellent source of independent review of a physics program, though they do not accredit individual physicists, nor does any currently existing program. Credentialing must be handled in‐house.

Solo physicists should carefully document all their work and create a clear archive of relevant information. This information will be crucial for credentialing a new physicist who will need to replicate the established processes in order to assure that continuity is maintained. This is clearly stated in the AAPM Code of Ethics:

“On leaving an institution, members have an obligation to leave all information for which compensation was made and to make a reasonable effort to facilitate an orderly transition of physics services. Documentation should be left in an intelligible, legible order and format. Materials generated as well as the notes from work compensated for by the institution are the property of the institution paying the salary or consulting fee of the individual doing the work. Such materials should be left in the possession of the institution unless otherwise instructed by the institution or agreed by the parties.”

Further issues with the solo practice of medical physics are discussed in AAPM Report Number 80.^(14)^

Regardless of the exact steps involved in learning a new procedure, the steps must be documented in detail. The revision dates of the policies and procedures, dates of performance, and proctor attestation of satisfactory performance should all be documented.

### 4. Credentialing and Ongoing Competency Evaluation of Existing Staff

When establishing a new competency and credentialing program where none had existed at an institution, the existing staff should have a baseline credentialing program to document their competence with current activities. This could include one or a combination of the following:

1. Observation of the performance of the task
2. Directed reading (policy and procedure, relevant literature) with or without knowledge test
3. Documentation of the prior completion of a minimum number of tasks

Once the initial credentialing has been documented, the ongoing competency of the staff should be evaluated.

Evaluation of these competencies can be done through audits of work product, direct observation of performance, self‐evaluation, or testing. Competency tests should emphasize knowledge, skills, and behavior. As with credentialing, this must be documented. The type and frequency of each competency must be detailed. The frequency should be determined by each facility but should be at least once per year. A sample list is shown in Table 1. This list is not intended to be exhaustive.

By collecting the documentation of these competency evaluations, some of the benefits that may be realized include:

1. The clinical physics program can be evaluated more easily.
2. The satisfaction of regulatory requirements can be demonstrated.
3. Individual staff members will have compiled individual portfolios which may prove helpful in subsequent licensing or credentialing activities.

It has been well documented that the quality of performance increases with greater repetition.^(15)^ In fact one study concluded: “The results of this study suggest that repetition may be more important than days since last trained for skill and knowledge retention.”^(16)^ Further, past mastery does not guarantee future competence without ongoing practice. To maintain competency with regard to a procedure, a minimum number of procedures must be performed each year. The number depends on the complexity and the frequency of the procedure. Each facility should determine its own requirements. A sample list of how this may be done is shown in Table 1. While this table lists annual requirements, other time frames may be used. For example, medical staff privileges generally run on a two‐ or three‐year cycle. Each facility should determine its parameters such that the facility is confident in the competence of the staff. If a staff member does not participate in the minimum number of procedures during a given time period, the individual must repeat the new staff credentialing process for that procedure. Mills^(17)^ has recently described such a program which can be used as a reference in developing a local program. To facilitate the tracking of who performs a procedure, the Electronic Medical Record (EMR) can be used. For example, the user who completes a task or approves a document is recorded by the EMR and a simple report can be run periodically to quantify how many procedures of each type are performed by the staff. Specifically in the Elekta Mosaiq EMR, Quality Checklist items can be created for typical physics work, such as IMRT QA, and Assessments can be created to document processes, such as second checks and weekly chart checks. A database query is then run to determine the user who approved or completed an item. Similar strategies can be used in Varian Aria.

**Table 1:** Sample list of competency assessments and minimum number of procedures performed per year

*Procedure*	*Type of Competency*	*Minimum Number of Procedures per Year*
Second check of treatment plan	Audit	12
Chart check	Audit	50
Patient specific IMRT QA	Audit	4
Treatment planning general	Audit	12
Treatment planning special procedure	Audit	4
Routine equipment QA	Observation	1
Output calibration	Outside audit (IROC or RDS)	1
Radioactive source strength calibration	Observation	1
Brachytherapy	Observation	4
Intraoperative radiation therapy	Observation	4
SRS/SBRT	Observation	4
Radiation Safety	Quiz	NA

While repetition is important in maintaining competency, objective feedback on performance is also important. One could repeatedly perform a task poorly and, without any feedback on performance, the task will continue to be done poorly. This is why annual competencies should involve observation, knowledge tests or audits, as described above, and mere repetition alone should not suffice to maintain competency.

### 5. Credentialing for New Technology

Implementing a new procedure or technology into a clinic should be a very deliberate process. All team members should be involved from the beginning. A sample process for implementation is shown in Appendix A. It is not intended to be prescriptive. Of the items listed, the department should retain documentation of training, policies and procedures, QA program development, and phantom testing. Any new procedure or technology is subject to Acceptance Testing and Commissioning by the QMP and this process should be documented in a Commissioning Report.

## IV. EVALUATION OF A COMPETENCY AND CREDENTIALING PROGRAM

To aid in the evaluation of a competency program, a guidance document should be prepared that lists each competency, the frequency of evaluation, and how it complies with relevant regulations or professional recommendations. Compliance with the department's program should be reviewed at least annually. This would involve verification that all staff members are current with required competencies and a review of the program to see if any changes need to be made. Sample documents are shown in Appendix B.

The program should be reviewed to ensure that it meets the requirements of institutional policy and national standards, such as those of The Joint Commission.

## V. FUTURE WORK

The methods and recommendations above should be regarded as examples only. Few appropriate studies have been published on the methods of establishing and maintaining technical competencies similar to those required in Medical Physics. A systematic study of these areas would be invaluable. One new methodology has recently been described by Brown et al.^(18)^ They describe a system to achieve competency in special procedures through a series of levels involving various instructional and evaluation techniques. This approach can possibly be expanded to include a wider range of activities.

## COPYRIGHT

This work is licensed under a Creative Commons Attribution 3.0 Unported License.

## APPENDICES

### Appendix A: Sample Implementation of New Technology Process

#### 1. Staffing

Before implementing a new procedure or technology, the facility should determine whether they have adequate staffing to support the activity, and if their current staff possess the appropriate experience and skills.

#### 2. Equipment

The QMP should determine if the appropriate dosimetry and test equipment is available for proper implementation of any new procedure or technology, as well as for continuing quality assurance.

#### 3. Training

Wherever possible, training by the vendor should be arranged. Ideally this would include off‐site teaching of the concepts involved and the use of the equipment and/or software, followed by on‐site training and observation of the first procedure. Staff attendance at nonvendor sponsored courses should be pursued whenever appropriate. All training must be documented.

#### 4. Policy and Procedure Development

All policies and procedures should be developed and reviewed by the clinical team and approved by the QMP and the Medical Director prior to the clinical implementation.

#### 5. Quality Assurance (QA) Program Development

The clinic's QA program must be updated to specifically address the newly introduced procedure or technology. Modifications to the QA program should be preceded by a thorough review of manufacturer's recommendations, professional society recommendations, applicable regulations, and the careful review of the entire process to find elements that require more rigorous testing. This could involve a formal Failure Modes and Effects Analysis (FMEA) process or other risk based methods.

#### 6. Dry Runs

Test runs of the entire process involving all team members should be performed. Careful attention to the written procedures should be given to identify any inaccuracies or items for improvement. These should be repeated until the entire team is comfortable, and should be documented. Simulation training involving the introduction of errors into the system to see how staff members respond is an evolving practice and is the topic of AAPM TG‐194 (in process). This could be a useful addition to the training process and could increase the level of safety.

#### 7. Phantom Measurements

Where applicable, ‘end‐to‐end’ tests with phantoms should be performed to evaluate the procedure or technology and its integration into the entire treatment process.

#### 8. Independent Review

Prior to clinical activation, the entire process in Steps 2–7 should be reviewed by a second physicist (internal or external peer, depending on group size). If the new technology involves radiation delivery, the output should have an independent validation using a dosimetry service or other method. Administrators and physicians should confirm that this review has taken place prior to the first clinical procedure.

#### 9. First Procedure

The first procedure should be performed with vendor support on‐site whenever possible. Arranging for an experienced proctor to be present on‐site would also be a reasonable approach.

#### 10. Postprocedure Team Meeting

After the first procedure, the entire team should meet to discuss any issues or suggestions for improvement.

#### 11. Ongoing Evaluation of Processes

As part of the policy and procedure development, a mechanism for the ongoing evaluation of the effectiveness of the procedure should be developed. For example, if a new SBRT program is started, appropriate metrics, such as planned dose gradients and shift data, should be monitored, as well as clinical outcomes.

### Appendix B: Sample Competency Program Documentation

#### 1. Competency Program Guidance Document

Date of last full review (document to be updated as new staff credentialed, full review indicates the annual review of competency details and verification of properly credentialed staff):

Competency program goals (see Table B.1):

1. Ensure proper credentialing of new staff
2. Ensure ongoing competence of all staff
3. Ensure safe implementation of new technology

**Table B.1:** Competencies

*Procedure*	*Related Regulations*	*Related Professional Recommendations*	*Method of Competency Evaluation*	*Minimum Number per Year To Maintain Competency*
Second check		AAPM TG40	Audit	12
Chart check		AAPM TG40	Audit	50
Patient specific IMRT QA		Guidance document on delivery, treatment planning, and clinical implementation of IMRT: Report of the IMRT subcommittee of the AAPM radiation therapy committee	Audit	4
Routine equipment QA	NY State part 16	AAPM Task Group reports 40,142	Observation	1
Output calibration	NY State part 16	AAPM TG51, AAPM Report 99	Outside audit	1
Radioactive source strength calibration		AAPM TG56	Observation	1
HDR	NY State part 16	AAPM TG59	Observation	4
Seed implants		AAPM TG56, AAPM Report 137, AAPM Report 128, AAPM TG64	Observation	4
Intraoperative radiation therapy		AAPM Report 152, AAPM TG72	Observation	4
SRS		AAPM TG42	Observation	4
SBRT		AAPM Report 101	Observation	4
Radiation Safety		AAPM Report 160	Test	NA

#### 2. Credentialed Staff Record

The department should keep an updated list of credentialed individuals. A sample list is shown in Table B.2.

**Table B.2:** Sample form for credentialized staff

*Procedure*	*Credentialed Staff*	*Date of Last Audit or Evaluation*
Second check		
Chart check		
Patient specific IMRT QA		
Routine equipment QA		
Output calibration		
Radioactive source strength calibration		
HDR		
Seed implants		
Intraoperative radiation therapy		
SRS		
SBRT		

#### 3. Staff Member Ongoing Competency Record

Competency can be assessed by auditing the records of previously performed procedures and via direct observation of a procedure (Table B.3).

**Table B.3:** Samples of staff competency record, audit form of previously performed procedures, and evaluation form

**SAMPLE STAFF COMPETENCY RECORD**					
Staff Name: John Doe					
Date of Hire: 1–1‐2008					
Competencies:					
*Procedure*	*Date Of Initial Credentialing*	*Previous Annual Competency Evaluations*	*Minimum Number/Year To Maintain Competency*	*Number Performed in Last Full Calendar Year*	*Records Attached*
Second check	1–10‐2008	1–30‐2010	12	200	Documentation of number of procedures and competency audit form.
Chart check	1–13‐2008		50	500	Documentation of number of procedures and competency audit form.
Patient specific IMRT QA	1–20‐2008		4	60	Documentation of number of procedures and competency audit form.
Routine equipment QA	1–30‐2008		1	6	Copy of QA records and competency audit form.
Output calibration	2–15‐2008		1	2	Outside audit results and copy of calibration reports
Radioactive source strength calibration	3–15‐2008		1	9	Copy of calibration report and competency evaluation form.
HDR	2–10‐2008		4	24	Documentation of number of procedures and competency evaluation form.
Seed implants	3–20‐2008		4	6	Documentation of number of procedures and competency evaluation form.
Intraoperative radiation therapy	4–15‐2008		4	2	Documentation of number of procedures and competency evaluation form.
SRS	9–1‐2008		4	6	Documentation of number of procedures and competency evaluation form.
SBRT	8–1‐2008		4	10	Printout from department information system documenting number of procedures and competency evaluation form.
Radiation Safety	1–4‐2008		NA	NA	Outline of safety in‐service and quiz result. DOT training documentation.

*Notes*: John Doe has not completed minimum number of IORT procedures in the last year. Must be re‐credentialed prior to performing independently.

**Table:** 

**SAMPLE AUDIT FORM**
Procedure
Items audited
Staff performing audit
Dates and number of procedures audited
Result of audit
Is staff member competent (yes/no)?
**SAMPLE COMPETENCY EVALUATION FORM**
Procedure
Items observed
Staff performing evaluation
Dates and number of procedures evaluated
Result of evaluation
Is staff member competent (yes/no)?
